# How to Best Name a Place? Facilitation and Inhibition of Route Learning Due to Descriptive and Arbitrary Location Labels

**DOI:** 10.3389/fpsyg.2016.00076

**Published:** 2016-02-01

**Authors:** Tobias Meilinger, Jörg Schulte-Pelkum, Julia Frankenstein, Gregor Hardiess, Naima Laharnar, Hanspeter A. Mallot, Heinrich H. Bülthoff

**Affiliations:** ^1^Human Perception, Cognition and Action, Max Planck Institute for Biological CyberneticsTübingen, Germany; ^2^Psychology of Education, University of MannheimMannheim, Germany; ^3^Cognitive Neuroscience, Department of Biology, Faculty of Science, Eberhard Karls University, TübingenTübingen, Germany; ^4^Department of Brain and Cognitive Engineering, Korea UniversitySeoul, South Korea

**Keywords:** spatial cognition, verbal overshadowing, dual coding, multimedia learning, wayfinding, virtual reality, primacy recency, orientation dependency

## Abstract

Establishing verbal memory traces for non-verbal stimuli was reported to facilitate or inhibit memory for the non-verbal stimuli. We show that these effects are also observed in a domain not indicated before—wayfinding. Fifty-three participants followed a guided route in a virtual environment. They were asked to remember half of the intersections by relying on the visual impression only. At the other 50% of the intersections, participants additionally heard a place name, which they were asked to memorize. For testing, participants were teleported to the intersections and were asked to indicate the subsequent direction of the learned route. In Experiment 1, intersections' names were arbitrary (i.e., not related to the visual impression). Here, participants performed more accurately at unnamed intersections. In Experiment 2, intersections' names were descriptive and participants' route memory was more accurate at named intersections. Results have implications for naming places in a city and for wayfinding aids.

## Introduction

Spatial information from a visible environment is processed not only (visuo)spatially, but also verbally: learning an environment while concurrently conducting a verbal secondary task was shown to influence performance in learning routes from video (Meilinger et al., 2008; Wen et al., 2011, 2012), from walking (Garden et al., 2002; Labate et al., 2014), from a map (Garden et al., 2002), as well as learning object locations in a room (Meilinger and Bülthoff, 2013). While verbal and non-verbal memory is involved in spatial learning, it is currently unknown how they interact during learning.

Paivio's dual coding approach (1971; 1986) provides a theoretical framework of how such a relation may look like. Dual coding states that verbal and non-verbal items are memorized in two separate, but corresponding memory systems. For example, a route walked may be memorized in a (visuo)spatial code which is also translated into a verbal route description. Both memory traces may be used for retrieval, and thus enhance retrieval performance. Such advantages have been repeatedly shown, for example, in verbal recall of single items (Paivio and Csapo, 1973), as well as for verbalizations in recognition of faces (Brown and Lloyd-Jones, 2005, 2006), drawings (Brown et al., 2014), pictures of mushrooms (Melcher and Schooler, 2004), and dynamic scenes (Huff and Schwan, 2008).

Corresponding memory traces can enhance recall. However, learning non-verbal material and forming a verbal memory trace of it has shown both enhancement, but also inhibition of learning, an effect called overshadowing. For example, Schooler and Engstler-Schooler (1990) showed participants a video with a person's face. Participants who verbally described the stimuli afterwards showed lower recognition performance than participants who did not do so. Beside face recognition (see Meissner et al., 2008 for an overview), this verbal overshadowing effect was found for routes presented on maps (Fiore and Schooler, 2002), color patches (Schooler and Engstler-Schooler, 1990), pictures of static scenes (Loftus et al., 1978), motor learning (Chauvel et al., 2013), wine tasting (Melcher and Schooler, 1996), as well as videos of social interaction (Adaval and Wyer, 2004), and dynamic scenes (Huff and Schwan, 2008). Overshadowing is not only observed after self-generated descriptions, but also after given ones (Dodson et al., 1997; Huff and Schwan, 2008).

To sum up, generating verbal memory traces for non-verbal material can facilitate or inhibit later recognition of the non-verbal material. According to the dual-coding approach, corresponding memory traces facilitate recognition. One explanation for inhibition through verbal overshadowing by retrieval-based interference proposes that descriptions induce a second memory trace different from the memory trace formed from the visual stimulus^1^. This second memory trace interferes with retrieving the first, original memory trace (Schooler and Engstler-Schooler, 1990). For example, the second, description based memory trace might be more similar to distractors and thus diminish correct recognition of the visual stimuli. Similarly, the two memory traces might render participants unsure which source to use afterwards (Dodson et al., 1997). Contrarily, when the description directly corresponds to the visual memory, participants show better recognition. Facilitation in visual recognition was observed under conditions which foster correspondence, namely when participants were instructed to use strict descriptions instead of lengthy ones (Meissner et al., 2001), when they were provided only with short rather than long time intervals for description (Brown and Lloyd-Jones, 2005, 2006), when they previously had received conceptual training rather than perceptual or no training in a formerly unknown domain (Melcher and Schooler, 2004), or when easy to name rather than difficult to name stimuli were used (Brown et al., 2014). Concise and informed verbalizations correspond more likely directly and thus lead to better performance. Lengthy descriptions might form correspondence as well, but they additionally may relate to different (visuospatial) representations (i.e., additional memory traces), which may also correspond to distractors and therefore yield lower performance. Huff and Schwan (2008) showed that recognition performance is enhanced if descriptions corresponded to later experienced visual material, but not to distractors. Their participants could align descriptions with the later perceived visual stimuli. However, when watching the visual stimuli first, later descriptions might not have corresponded to verbal memory traces formed before and thus interfered with each other yielding overshadowing.

The crucial point is that when descriptions and visual stimuli closely correspond facilitation is observed. If no such close correspondence is established or non-exactly corresponding additional verbal memory traces are formed, overshadowing may occur. The motivation for the present work was to examine whether effects of facilitation and overshadowing also generalize to a domain with a high everyday relevance namely wayfinding. In our experiments, participants walked a predefined route and were asked to remember the intersections that either had labels or no labels. In Experiment 1, we used arbitrary non-meaningful labels, in Experiment 2 corresponding descriptive labels. The arbitrary non-meaningful labels provided no correspondence between the verbal label and visual stimuli. We predicted overshadowing (i.e., worse performance) in learning with label as compared to learning without label. Descriptive location labels of Experiment 2 corresponded to the visible intersection. We predicted facilitation (i.e., better performance) as compared to learning intersections without label.

## Experiment 1

### Materials and methods

#### Participants

Nineteen participants (10 female, nine male; age: *M* = 26; *SD* = 4.3) were recruited via a subject database, gave written informed consent and were paid for their participation. Fifteen participants performed better than the chance level of 67% errors (*p* < 0.08) and were thus included into the analysis. This research was approved by the ethical committee of the university hospital of Tübingen.

#### Materials

Participants' head position was tracked by 16 high-speed motion capture cameras at 120 Hz (Vicon® MX 13) while they walked freely in a large tracking space (15 × 12 m), experiencing a virtual maze. The participants' head coordinates were transmitted via wireless connection (using WLAN) to a notebook computer (Dell XPS M170) which was mounted on a backpack, carried by the participant. This notebook rendered an egocentric view of the virtual environment in real-time using a NVIDIA GO 6800 Ultra graphics card with 256 MB RAM. Participants viewed the scene using a light-weight stereo head-mounted display (HMD; eMagin Z800 3D Visor) that provided a field of view of 32 × 24 degrees at a resolution of 800 × 600 pixels for each eye. The overall setup provided important depth cues such as stereo vision and motion parallax, as well as all bodily cues important for orientation including efference copy, vestibular and proprioceptive information.

**Figure 1:**
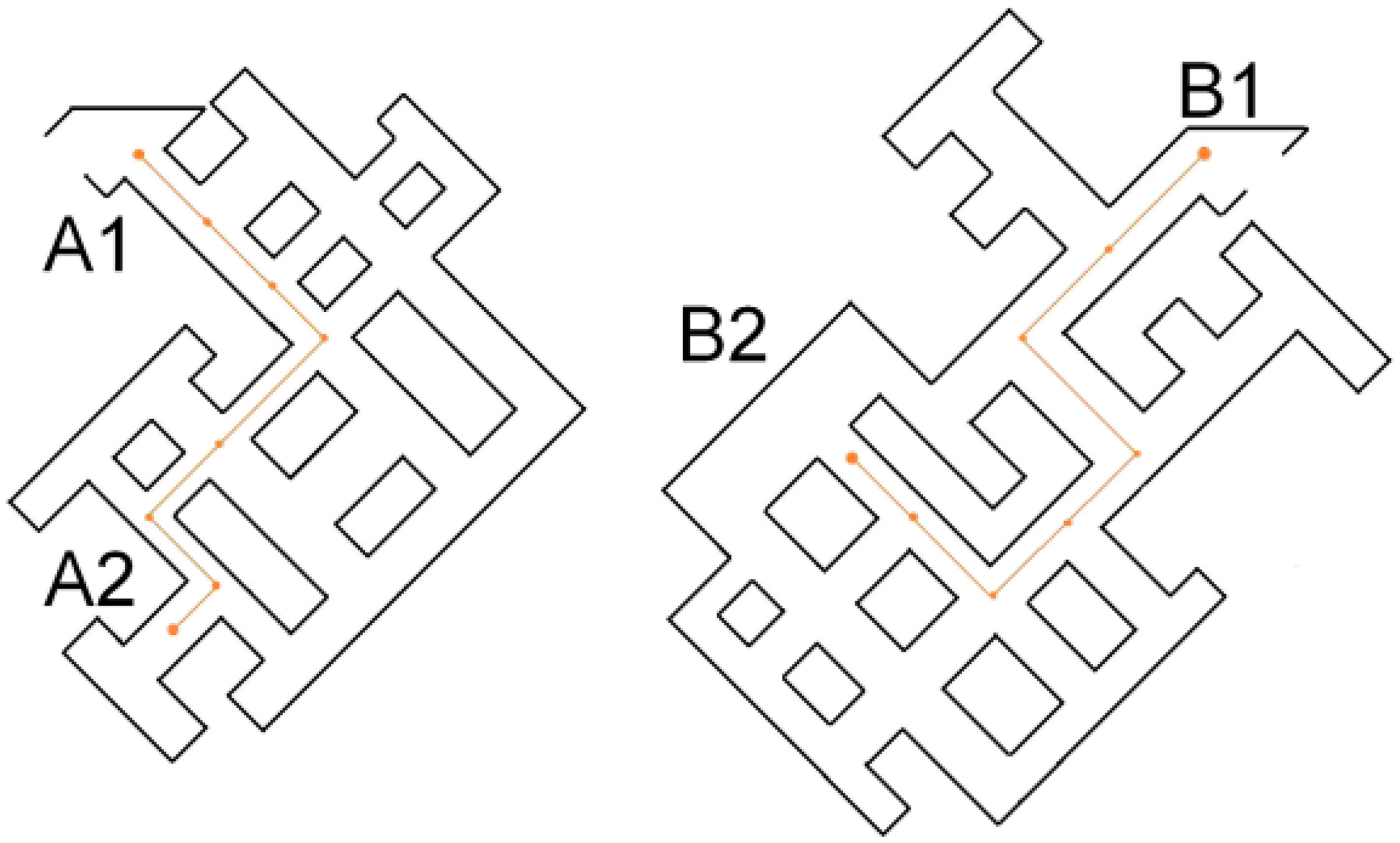
**Maps of route A (left) and route B (right) which each participant learned by walking**. On route A participants either started from A1 or from A2 (accordingly B1 or B2 for route B). Note that the corridors were all of different widths, so each junction was unique in geometry. However, the more salient difference between intersections was coded by textures.

**Figure 2:**
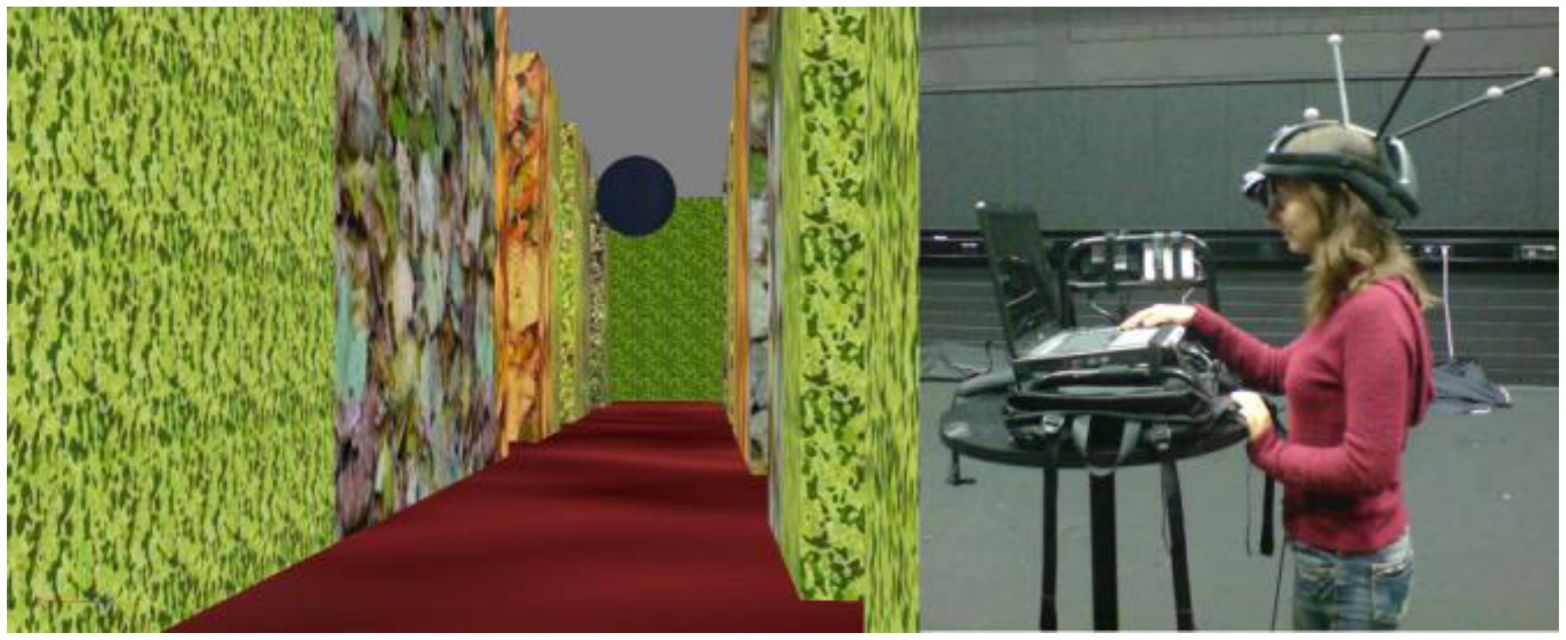
**Experimental setup**. Left side: Participants were guided through the maze by a sphere floating at 1.6 meters height. The sphere stopped at each junction for 20 s. Right side: A participant during the test phase, equipped with a tracking helmet and head-mounted display (HMD). During the experiment the lights were turned off and the head was covered with additional blinds.

The experiment was programmed in Virtools 5.0 (Dassault Systems®). The two virtual mazes that participants experienced via HMD were modeled in such a way that all junctions in both mazes were rectangular (see Figure 1), but differed in geometries (i.e., corridor widths and lengths) and textures (see Figure 2). Textures contained leaves and plants.

#### Procedure

The experiment was separated into a learning phase and a test phase. In the learning phase, participants were asked to walk a specific route through the virtual maze twice. They were guided by a sphere floating at 1.6 meters height which stopped in the middle of each junction they passed (see Figure 2). When the sphere stopped, participants either heard the name of the intersection or an instruction to remember the intersection (based on visual cues). After 20 s the sphere would begin to move again, either in the same direction (three intersections) or in a different direction (three intersections; see Figure 1). Participants were instructed to remember an intersection either based on what they could see (and not to generate a name for the intersection) or to remember the intersection as well as the label assigned to it.

Three intersections on each route were selected randomly for each participant and were associated with a label. The other three intersections remained unlabeled. The labels were arbitrary and did not correspond with the botanic texture or the geometry of an intersection (e.g., “Berliner Platz,” “Goethe Platz”). After the first walkthrough the display turned black and participants were guided by the experimenter back to the start location from which they started a second learning run along the same route, before proceeding to the test phase.

In the test phase, participants were standing in front of a high table (see Figure 2), wearing the HMD through which they experienced an egocentric view of one of the intersections (without the sphere). The table was placed in the middle of the (virtual) intersection, so that participants got the impression of approaching the intersection from one of the corridors. Participants were allowed to move their head and look about, but were asked not to walk around.

First, participants were asked to identify as quickly and accurately as possible the direction they had walked during the learning phase after leaving that particular intersection. They responded by pressing one of the four arrow keys on the laptop keyboard. We recorded the time between the presentation of the intersection and the key press. Selecting the correct route alternative was considered a hit, selecting one of the alternatives an error. We defined chance level as randomly guessing between three route alternatives which yields a hit rate of 33% or a respective error rate of 67%. Subsequently, participants were asked whether this particular intersection was labeled. If they indicated that the intersection was labeled, they had to identify the correct label from a list of six options. This list contained the three labels used on their route and three unknown distractors the participant had not heard previously in the experiment.

Participants were tested on all perspectives one could approach a junction from a corridor, they were never tested with a wall at their back. This resulted in 19 test trials for route B and 20 for route A (Figure 1).

The learning and test phases for each route followed on immediately from one another (the learning phase for route A was immediately followed by the test phase for route A etc.). The presentation order of routes A and B was balanced across participants, as were the walking directions (routes started at A1&B1, A2&B2, B1&A2, or B2&A1).

#### Analysis

For the analysis, we removed data deviating more than three standard deviations from the overall mean. We conducted a linear mixed model analysis (Snijders and Bosker, 1999) with the random factor participants and fixed factors label (yes/no), order of presentation (four orders), intersection number (1–6), and perspective (experienced along the route/other) including all possible interactions (full factorial design). The order of presentation and the number of intersections were included for control reasons. Significant interactions with labeling never changed the main effect of labeling and are therefore not further reported. Perspective was of interest, as route directions are mainly uttered in the orientation of walking a route (Daniel and Denis, 2004). Furthermore, intersections were shown to be memorized within the experienced perspective (Meilinger et al., 2012). Commonly accepted effect sizes for linear mixed models are not yet available. We thus report Cohen's *d* and partial eta square η${}_{p}^{2}$ derived from data aggregated per participant and the respective condition.

### Results and discussion

**Figure 3:**
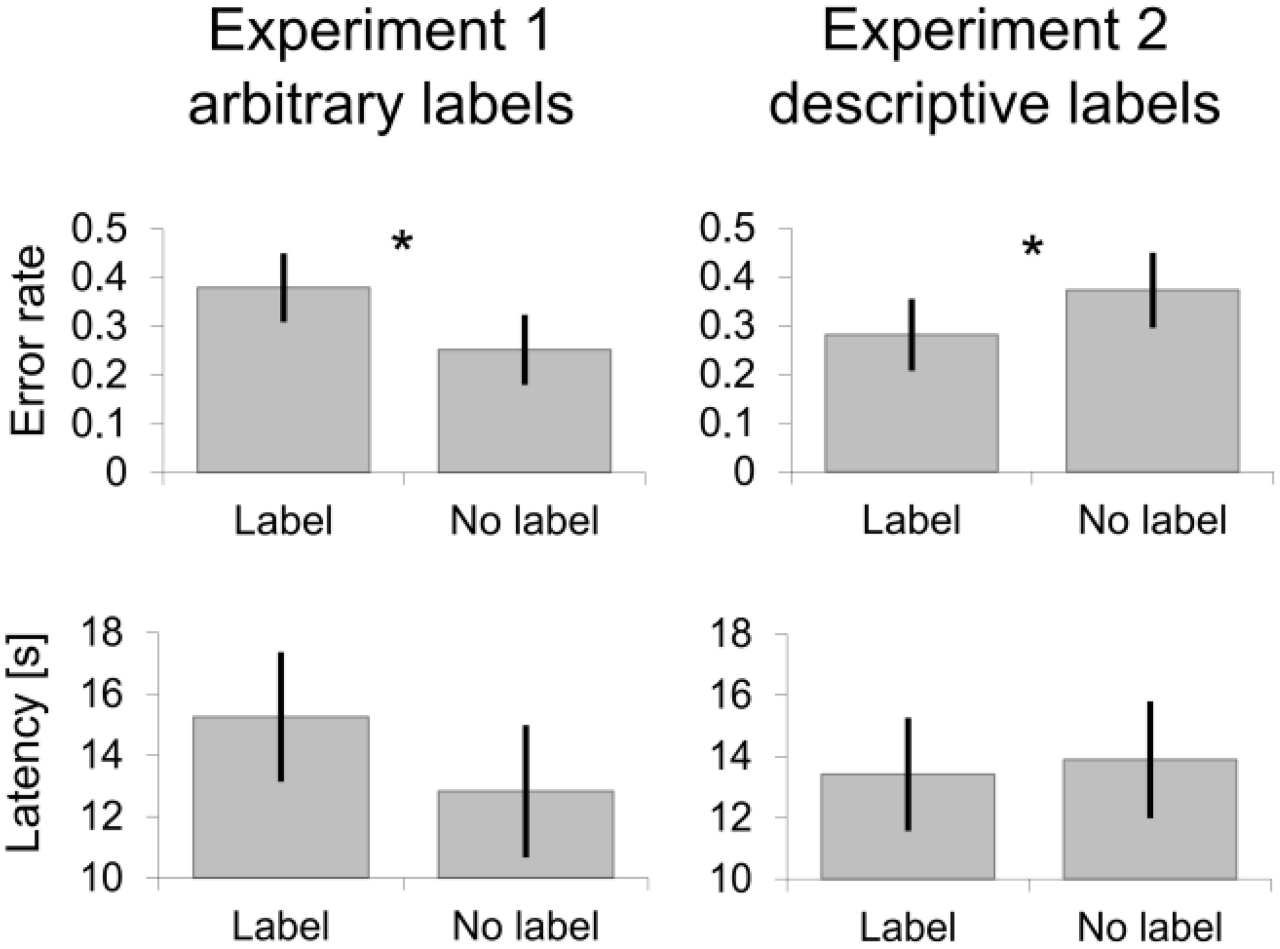
**Wayfinding performance at intersections with and without labels**. Means and standard errors as estimated from the marginal means are shown. Asterisks ^*^ mark significant differences.

We expected that a lack of correspondence between arbitrary labels and representations of intersections inhibited route learning. As predicted, participants were more accurate, *F*_(1, 494)_ = 4.26, *p* = 0.040, *d* = 0.74, and by trend faster, *F*_(1, 479)_ = 3.61, *p* = 0.058, *d* = 0.67, in indicating the route at non-labeled intersections (Figure 3 left side).

Participants reacted faster at intersections presented in the same perspective and walking direction as experienced in the learning phase (*M* = 12.7 s, *SE* = 1.1 s), compared to all other viewing directions (*M* = 15.4 s, *SE* = 1.0 s), *F*_(1, 478)_ = 9.86, *p* = 0.002, *d* = 1.17. This orientation dependency in memory for spaces is well-established in the literature for objects (Bülthoff and Edelman, 1992), room-like spaces (Diwadkar and McNamara, 1997), as well as locations in environmental spaces such as buildings or cities (Christou and Bülthoff, 1999; Meilinger et al., 2012). The test perspective did not interact with labeling accuracy, *F*_(1, 491)_ = 2.16, *p* = 0.142, or latency, *F* < 1.

**Figure 4:**
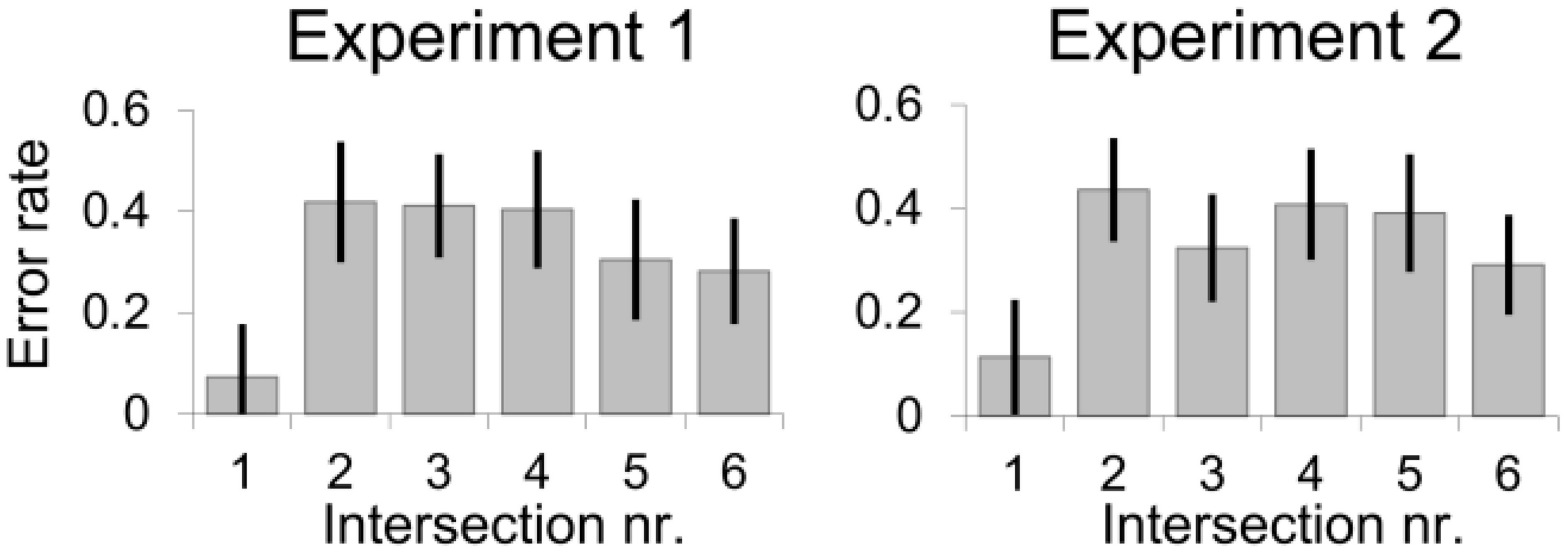
**Primacy (advantage for first items) and potentially recency (advantage for last items) effects in route memory**. Means and standard errors as estimated from the marginal means are shown.

We also observed a primacy and maybe also a recency effect for the error rate data. Figure 4 displays the averaged data of all six intersections in the order of presentation. As depicted, intersections close to the start or end of the route were remembered best, while performance was worse for the intersections in between, *F*_(5, 494)_ = 5.58; *p* < 0.001, η${}_{p}^{2}$ = 0.16. The primacy and recency effects were also found in another route learning experiment with children (Cornell et al., 1996) and suggest that route learning follows similar laws known from learning verbal lists (Postman and Phillips, 1965).

Based on considerations from dual coding and verbal overshadowing we proposed that a lack of correspondence between the visual stimulus and the verbal label as instantiated with arbitrary location labels resulted in worse performance as was observed. Within the context of overshadowing a verbal label may trigger a memory different from the non-verbal memory which interferes with recognizing the visual stimulus (Schooler and Engstler-Schooler, 1990; Huff and Schwan, 2008). Perceptual associations to arbitrary labels like “Berlin” had no relation to any of the textures used. Therefore, interference may have caused the performance drop observed.

There is also another interpretation for this effect based on multimedia learning (Sweller et al., 1998; Paas et al., 2003; Mayer, 2009). When learning multimedia stimuli, for example, pictures and texts together, both media profit from each other in the sense of Paivio's dual coding only if they correspond to each other. The text has to relate to the picture conceptually. If this is not the case, they are not “coherent” which inhibits learning as we observed (Mayer and Jackson, 2005). A related conception refers to “extraneous load” which inhibits learning (Sweller et al., 1998; Paas et al., 2003). Our participants had to learn the route, but at certain intersections they were additionally required to learn arbitrary labels for them. This label learning task was extraneous to the route learning task and therefore inhibited route learning as observed. Coherence, extraneous load, and interference are all valid explanations for the effect observed.

In addition to the effects mentioned it is also possible that participants in the no-label condition generated labels by their own—even or especially as they were asked not to do so. Corresponding self-generated labels might have helped participants memorizing intersections in the sense of dual-coding. The crucial point is that non-corresponding labels had detrimental effects relative to that.

The present data shows, that inhibition due to verbal learning generalizes to the field of wayfinding and navigation. However, the inhibition of route learning is only one part of the story, in order to tell the whole story also facilitation of route learning due to a correspondence between intersection and label must be shown. This is the motivation for Experiment 2.

## Experiment 2

Results from Experiment 1 showed that learning arbitrary location labels interfered with route learning. In Experiment 2, we used descriptive labels (i.e., an ivy-textured intersection was labeled “ivy place”). These descriptive labels were thought to connect the memory for an intersection and a label and therefore enhance route learning.

### Materials and methods

#### Participants

Thirty-four participants (19 females, 15 males; age: *M* = 24; *SD* = 4.6) were recruited via a subject database, gave written informed consent and were paid for their participation. Twenty-four performed better than chance level of 67% errors (*p* < 0.05) and were thus included into the analysis.

#### Materials and procedure

We used the same setup and procedure as in Experiment 1 except for the following change. In order to provide descriptive labels for intersections, we asked 15 participants who did not participate in Experiments 1 or 2 to name printouts of textures used in the environment. Two raters selected the 12 textures which were named most unequivocally, for example, grass, ivy, moss, clover, bark, hay. These textures which had been located at various locations in the environment in Experiment 1 were then placed at intersections along the routes. Textures located along the route in Experiment 1 were moved to the former locations of these 12 selected textures (i.e., to intersections typically not located along the route in Experiment 1). Consequently, the same textures were used for the environment of Experiment 2, but 24 textures were changed in their locations. Procedure and analysis were identical to Experiment 1.

### Results and discussion

Data showed a main effect of labeling for accuracy in route selection, *F*_(1, 793)_ = 4.08; *p* = 0.044, *d* = 0.33, and no effect in time, *F* < 1. As predicted, participants performed better at labeled intersections. With descriptive labels and thus with presumably already established connections between visuospatial and verbal memory better memory for route directions was observed.

Figure 4 shows the effect of intersection number on accuracy, *F*_(5, 796)_ = 6.20; *p* < 0.001, η${}_{p}^{2}$ = 0.24, suggesting a primacy and perhaps also a recency effect. As in Experiment 1, participants reacted faster when intersections were presented in the same perspective as experienced in the learning phase (*M* = 12.3 s, *SE* = 0.94 s) compared to all other viewing directions (*M* = 14.7 s, *SE* = 0.84 s), *F*_(1, 777)_ = 12.81, *p* < 0.001; *d* = 0.95. Test perspective did not interact with labeling, *F*'s ≤ 1.

The performance advantage for intersections with descriptive labels suggests that navigators relied on existing correspondence between visuospatial and verbal memory and did not do so when learning without labels. One interesting question asks which the default case of route learning is: visuospatial only learning (i.e., without verbal memory trace) or learning visual and verbal learning together? If participants naturally learned routes without generating verbal labels, then the two no-label conditions of Experiment 1 and 2 should show similar performance levels. However, this is not obvious in the data. As indicated in Figure 3 learning without (arbitrary) label in Experiment 1 seems similar to learning with descriptive label in Experiment 2. And learning an arbitrary label in Experiment 1 resembled the error rate of learning without a (descriptive) label in Experiment 2. We think that this inconsistency is resolved when assuming default route learning as dual-coding verbal and corresponding visuospatial memory traces. This assumption is supported by secondary task experiments which show that visuospatial and verbal memory traces are both involved in route learning (Garden et al., 2002; Meilinger et al., 2008; Wen et al., 2011, 2012; Labate et al., 2014). In Experiment 2 participants used corresponding memory traces when learning the route with descriptive labels. Suppressing descriptive label usage inhibited performance relative to that. In Experiment 1 the deviation from normal route learning was when participants learned arbitrary non-corresponding labels. It might be that participants self-generated corresponding labels in the no-label conditions, interpreting the instruction in a sense of not using any arbitrary labels rather than not using labels at all. In Experiment 2 this is unlikely as labels were descriptive and not using descriptive labels means using no labels at all rather than using arbitrary labels instead. In that interpretation employing given or self-generated descriptive labels was a rather natural situation and yielded similar good performance. Deviating from that situation by learning arbitrary labels or by inhibiting descriptive labels degraded performance relative to that. Please note that this interpretation of the data still supports the basic assumption that correspondence between verbal and visual memory traces facilitates and non-correspondence inhibits performance.

## General discussion

The present experiments show that facilitation and inhibition effects due to verbal processing in a non-verbal task (e.g., Schooler and Engstler-Schooler, 1990; Huff and Schwan, 2008; Meissner et al., 2008) also occur in the domain of wayfinding. This connects literatures of verbal overshadowing and multimedia learning with wayfinding, showing that their findings are also important in everyday environmental learning. Verbal processing during spatial learning can have auxiliary and detrimental effects as a function of how verbal and visual stimuli correspond.

These findings have practical implications for location naming as well as route directions. If possible, place names should semantically correspond to their named location or locations should not be labeled at all. “Goethe plaza” as a name for a plaza without any reference to this writer (e.g., a statue) is an arbitrary name. It does not correspond to anything seen there and based on findings of Experiment 1 one can expect navigators to learn such a location better without any name rather than an arbitrary name. At least navigators are able to construct descriptive labels by their own. If a location has to be named a descriptive name is advisable. The labels in Experiment 2 were obtained from consensual descriptions of what could be seen at a location and participants performed better at labeled than unlabeled intersections. Relying on a correspondence between the verbal label and visual features will help participants' memory compared to a situation where such a correspondence is simply not established or actively suppressed. We do not know whether given descriptive labels are better than self-generated ones—they probably are not. But if location labels have to be established as typically is the case for most city locations it is advisable to try using descriptive ones. Descriptive names may also support wayfinding aids. Useful route directions identify decision points and subsequent route decisions (Denis et al., 1999). Locations may be recalled best from directions in which location names correspond to what can be seen at this location. This effect may apply for verbal directions as well as for satnavs and thus provide a means to improve wayfinding.

Beside facilitation and inhibition, primacy and maybe also recency effects, i.e., better memory for items at the beginning and end of a sequence, were observed. These effects are known in learning lists (Postman and Phillips, 1965). Together with a study from Cornell et al. (1996), we show that primacy/recency effects also generalize to learning routes. Just like lists, routes are ordered sequences of elements learned from start to the end.

Facilitation and inhibition effects with labels fit the theoretic framework which extends Paivio's dual coding approach (1971, 1986) toward verbal overshadowing (e.g., Schooler and Engstler-Schooler, 1990; Meissner et al., 2008) as well as multimedia learning (Sweller et al., 1998; Paas et al., 2003; Mayer, 2009). Correspondence between verbal and non-verbal memory traces by learning meaningful labels facilitates later retrieval. Contrary, if such connections do not exist as in the case of arbitrary labels retrieval is inhibited. Several explanations for inhibition are possible within this framework. Inhibition could originate from source interference between memory from the non-verbal stimuli and memory from the verbal stimulus. However, in our experiment, this interference was definitely not based on a higher similarity of the verbal memory trace with a distractor (Huff and Schwan, 2008) as we did not use a recognition task, and the arbitrary labels had no similarity to any texture used. Also, the explanation of being unsure about the relevant memory trace (Dodson et al., 1997) is unlikely as participants in our experiments had to act upon their visual input during testing. An interpretation based on extraneous load (Sweller et al., 1998; Paas et al., 2003) seems most plausible to us. Learning connections between arbitrary labels and intersections in addition to the route poses additional load to a resource limited system and thus inhibits route learning at these intersections. Connections to descriptive labels do not have to be learned and at these locations learning profits from dual coding. Irrespective of the exact mechanism for inhibiting route learning in Experiment 1 (i.e., source interference or external load), both rely on separate memory traces which enhance performance when connected and which interfere with route learning if such a clear mapping is not present. In the end the current data do not allow for strong conclusions regarding the mechanisms of inhibition. The main point made here is that inhibition effects have to be considered also in the context of wayfinding.

Experiments with children learning feature—location combinations on figures (Dessalegn and Landau, 2008) showed that children profited from descriptive hints such as “the red is on the left,” but not so from arbitrary labels “this is a dax” or task irrelevant descriptions like “the red is touching the green.” Similarly, children and adults exhibited better route memory when landmarks had an additional corresponding label than without label (Lingwood et al., 2015). These experiments showed facilitation for task-relevant descriptions, but did not show inhibition due to arbitrary labels as our experiments did.

Familiarity with the descriptive labels in contrast to unfamiliarity with the arbitrary labels (Sloutsky and Robinson, 2008) cannot explain the present results. The arbitrary labels such as “Berlin place” were not fantasy words never been heard before. In fact, they are even mentioned more often in electronic magazines and newspapers than the descriptive labels (Quasthoff et al., 2006). Arbitrary labels merely did not correspond to what could be seen at an intersection.

An interesting addition to prior studies is that the task used was no mere recognition task. Participants had to indicate route continuation. Recognizing an intersection was not sufficient for this task. Participants who recognized intersections, but guessed route continuation, were excluded from the analysis. Facilitation and inhibition of route retrieval must have encompassed a conglomerate of location memory and further route continuation. Please note that participants could not have simply remembered textures along the route where to walk toward. If this was the prevalent strategy, then no effect of labeling could have been observed, as labels were assigned to the current intersection, not to a distant one.

The observation that spatial learning incorporates both inhibition and facilitation through verbal memory has consequences for future experiments within spatial memory research. Memory for non-verbal stimuli can differ considerably depending on whether participants verbalize the stimulus material and the way they do it. For example, the length of verbalizing and the expertise may inhibit or facilitate later retrieval (Meissner et al., 2001; Melcher and Schooler, 2004; Brown and Lloyd-Jones, 2005, 2006). The present results suggest that this has to be considered when examining spatial memory, especially as verbal memory for spatial locations cannot only facilitate or inhibit recall, but also has the potential to alter the structure of the recalled memory (Shelton and McNamara, 2004; Meilinger and Bülthoff, 2013). Therefore, future experiments have to be sensitive to what extend verbal memory traces may affect the results obtained and whether results can be generalized to other learning situations with more or fewer opportunities for verbal processing.

## Conclusions

Present results indicate that verbal facilitation and inhibition of non-verbal memory known from overshadowing and multimedia learning also extends to wayfinding. Place names corresponding to what can be seen at a location facilitate learning and may thus be considered when naming places and constructing wayfinding aids.

## Author contributions

All authors designed research; TM, JS, JF, and NL performed research and analyzed data; all authors wrote the paper.

### Conflict of interest statement

The authors declare that the research was conducted in the absence of any commercial or financial relationships that could be construed as a potential conflict of interest.

## Footnotes

^1^For face recognition and visual imagery, also a shift between holistic and feature processing was proposed. However, this difference is difficult to apply to motor learning, color patches or, as in the present case, wayfinding.
